# Symptom distress evolution over the first year after allogeneic stem cell transplantation – a prospective observational sub-study of the SMILe project

**DOI:** 10.1007/s00520-026-10546-9

**Published:** 2026-03-17

**Authors:** Anja Schmid, Janette Ribaut, Sabina M. De Geest, Sabine Valenta, Robert Zeiser, Kris Denhaerynck, Klaus Kaier, Alexandra Teynor, Lynn Leppla

**Affiliations:** 1 Department of Medicine I, Faculty of Medicine, Medical Center – University of Freiburg, Hugstetter Str. 53, D-79106 Freiburg im Breisgau, Germany; 2 Department of Nursing, Medical Center – University of Freiburg, Freiburg im Breisgau, Germany; 3 Institute of Nursing Science (INS), Department Public Health, University of Basel, Basel, Switzerland; 4 Academic Center for Nursing and Midwifery, Department of Public Health and Primary Care, KU Leuven, Louvain, Belgium; 5 Practice Development and Research Division, Medical Directorate, University Hospital Basel, Basel, Switzerland; 6 Institute for Medical Biometry and Statistics, Faculty of Medicine, University of Freiburg, Freiburg im Breisgau, Germany; 7 Faculty of Computer Science, Technical University of Applied Sciences Augsburg, Augsburg, Germany

**Keywords:** Allogeneic stem cell transplantation, Observational studies, Electronic patient reported outcome measures, Symptom distress

## Abstract

**Purpose:**

Symptom-related distress after allogeneic stem cell transplantation (alloSCT) significantly impairs quality of life and long-term health. However, symptom distress evolution is not substantiated and potential influences of patient-related factors remain unclear. This study's aims were to (A1) describe first-year post-alloSCT symptom distress evolution, (A2) explore that evolution in view of three patient factors: graft-versus-host-disease (GvHD), gender, age group.

**Methods:**

This prospective longitudinal observational sub-study of the main SMILe (SteM cell transplantatIon faciLitated by eHealth) study used demographic and clinical data of patients receiving the SMILe Integrated Care Model, and their electronic patient-reported symptom distress outcomes. Patients reporting ≥ 1 time pre- and regularly post-alloSCT on 10 relevant symptoms (distress = 1–10 scale, none = 0), were included. We calculated distress point prevalences at 10 time points and plotted distress score trajectories, determining distress durations and distress persisting > 30 days uninterrupted throughout the first year post-alloSCT (A1). We used boxplot faceting visualising distress persistence by patient factors, and sought parallel-evolving GvHD and distress point prevalences (A2).

**Results:**

Four symptoms contributed to the highest distress point prevalences (fatigue 48.2–76.9%, decreased appetite 21.7–56.9%, pain 25.4–47.7%) and high-scoring distress trajectories (≥ 3 in fatigue, decreased appetite, dyspnea) – with months of uninterrupted distress (65.7% of patients). We identified at-risk patient groups with exceptional distress persistence. Parallel-evolving point prevalences from day + 90 post-alloSCT suggested GvHD influenced symptom distress.

**Conclusion:**

Symptom-related distress monitoring and management in alloSCT follow-up is important. GvHD management may require gender- and age-tailored interventions against distress persistence.

**Trial registration:**

The main SMILe study was registered at who.int/clinical-trials-registry-platform: DRKS00020347, https://trialsearch.who.int/Trial2.aspx?TrialID=DRKS00020347, date of registration: 03/01/2020 (Freiburg) and clinicaltrials.gov: NCT04789863, https://clinicaltrials.gov/expert-search?term=NCT04789863, first posted: 03/10/2021 (Basel).

## Introduction

Following allogeneic stem cell transplantation (alloSCT), patients’ first years are commonly impacted by complications such as relapse, infections, and graft-versus-host-disease (GvHD) [1]. Particularly, GvHD affects multiple organs with variably severe symptoms [2, 3]. Regarding the potential severity of non-relapse complications (e.g. GvHD, infections) [1–3], early recognition can accelerate treatment initiation and improve treatment response [4, 5]. However, recognition relies on monitoring of signs and symptoms, conventionally demanding resource-intensive in-person contact. An efficient complement to routine monitoring is remote patient-reported monitoring, enabling early symptom management. Recent research has shown excellent potential of remote monitoring to reduce risks and perceived burdens of non-relapse complications including acute health deterioration, lowered quality of life (QoL), frequent hospital readmissions, and increased mortality [6–8].

Enhancing patient outcomes depends on comprehensive knowledge about post-alloSCT symptoms for optimised symptom management. Symptoms have an impact on outcomes and can vary over time, e.g. through interventions [9]. They can be measured in two dimensions: *occurrence* (e.g. severity, frequency) and *distress* (i.e. discomfort related to symptoms) [10]. Although post-alloSCT symptom *occurrence* is adequately described [11–22], research on symptom *distress* is limited [18–24]. High symptom distress significantly reduces QoL [19, 23] and, at day + 120, significantly contributes towards health impairments at 1 year post-alloSCT [22]. Associations with impairments remain up to 14 years [21–23], potentially impacted by patient-related factors (e.g. GvHD [24], gender [25], age group [19]). This raises important questions about the persistence of elevated symptom distress and the patient-related factors influencing it. However, current knowledge on post-alloSCT symptom distress evolution remains insufficient, and full first-year trajectories are still not described. Previous studies used short observation periods (e.g. ≤ six months [18–21]), few measurement points (e.g. ≤ three times/year post-alloSCT [22]), or symptom questionnaires lacking alloSCT-specific validation [18, 19, 23, 24]. In view of these limitations, a more detailed understanding of post-alloSCT symptom distress evolution and the ability to identify patient subgroups at heightened risk is essential for developing targeted supportive interventions. Accordingly, we aimed to (1) describe symptom distress evolution over the first year post-alloSCT, and (2) explore symptom distress evolution in view of GvHD, gender, and age group.

## Methods

### Design, setting and sample

This longitudinal observational sub-study used prospective data from a randomised controlled trial evaluating the **S**te**M** cell transplantat**I**on faci**L**itated by **e**Health–Integrated Care Model (SMILe–ICM) in Freiburg, Germany, and Basel, Switzerland [6, 26–32]. It was approved by the ethics committees of Freiburg (EK 309/19) and Northwestern & Central Switzerland (EKNZ 2021–00202). All patients provided informed written consent for pseudonymised data collection and analysis. Recruitment, follow-up, and data collection lasted from 2/2020–8/2022 (Freiburg) [6] and 4/2021–9/2023 (Basel) [27].

**Fig. 1:**
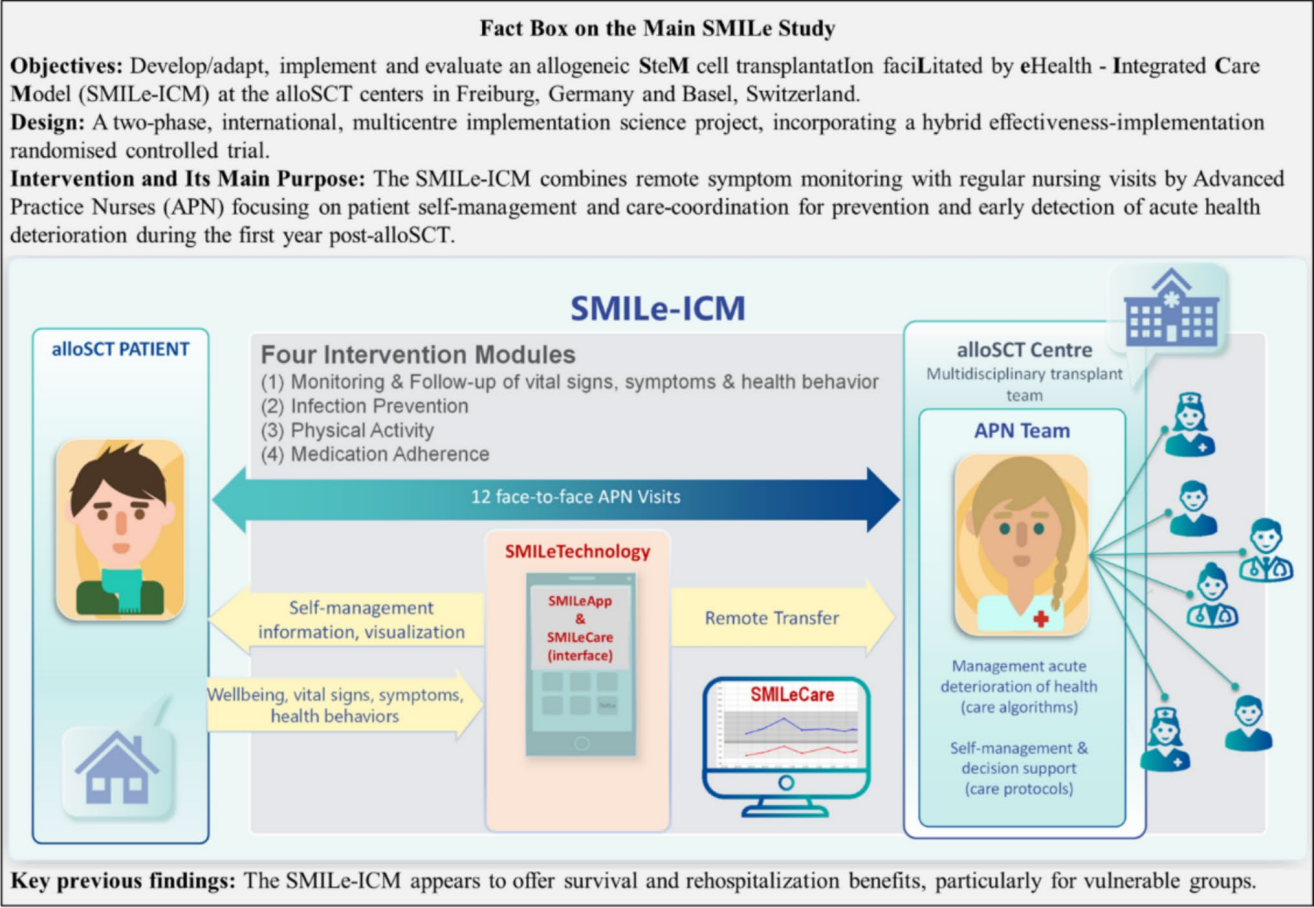
Main SMILe Study Fact Box

The main SMILe study tested the SMILe–ICM intervention versus usual care [6, 27]. From day −10 (± 7) pre-alloSCT (enrolment) through day + 365 (± 7) post-alloSCT, SMILe–ICM combined regular face-to-face interventions by advanced practice nurses with the SMILe patient self-management technology [6, 27–30]. Additionally, remote monitoring via SMILeApp enabled rapid care team responses to patient-reported parameters and symptoms [29], see Fig. 1 for a main SMILe study fact box. Details on study procedures and SMILe–ICM are reported elsewhere [6, 26–32].

**Fig. 2:**
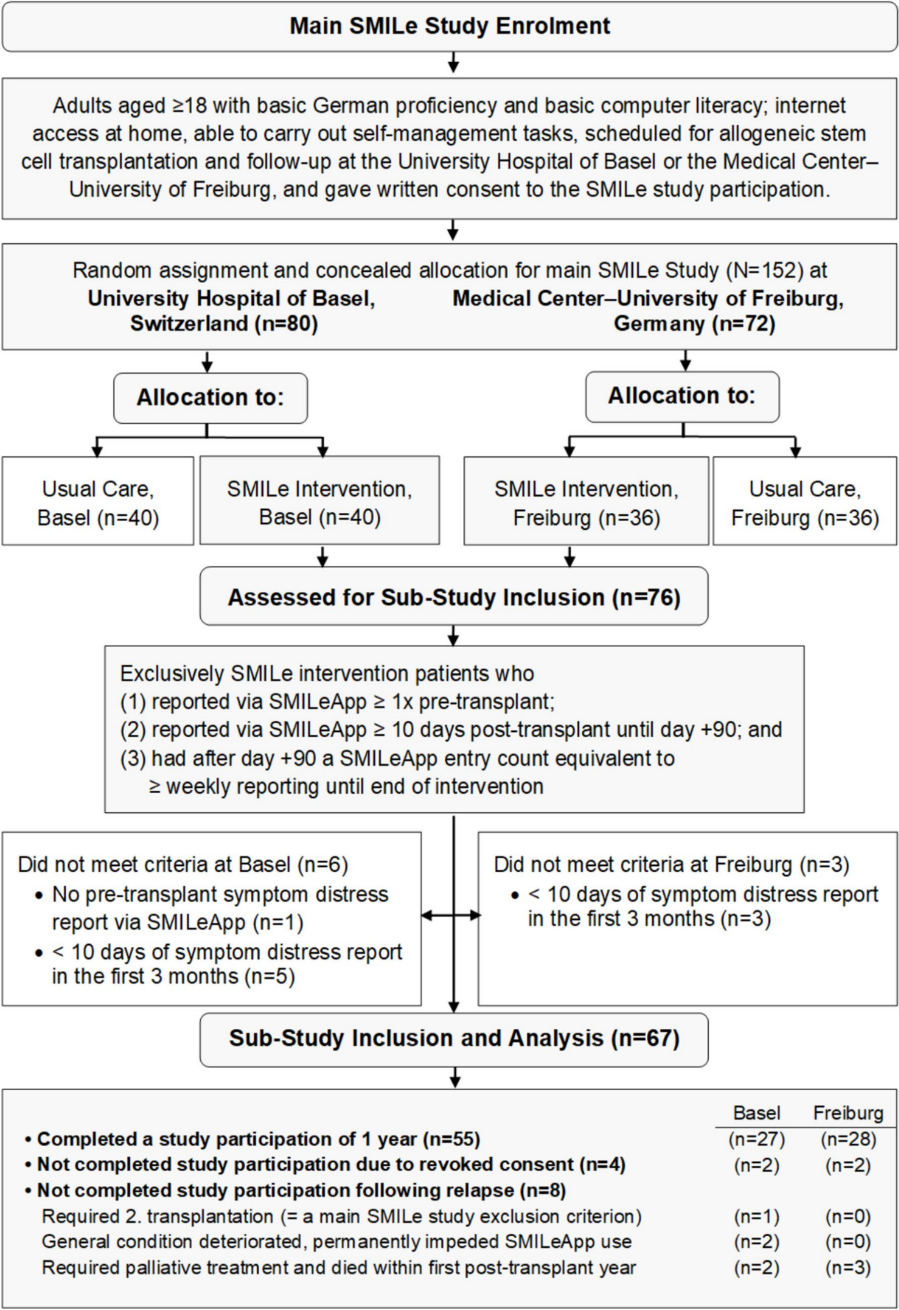
Overall SMILe Flow Diagram From Main SMILe Study and This Sub-Study

For this sub-study, we reviewed all SMILeApp entries from both the Freiburg and Basel intervention groups, applying the following inclusion criteria: alloSCT patients aged ≥ 18, who reported via SMILeApp (1) ≥ 1 time pre-alloSCT, (2) ≥ 10 times post-alloSCT until day + 90, and afterwards had (3) a minimum SMILeApp entry count equivalent to weekly reporting until the intervention’s end. Figure 2 shows the patient flow in the main SMILe study and this sub-study.

### Variables and measurements

Patient-related variables at enrolment included *gender, age, living alone, diagnosis of underlying disease*, and *conditioning treatment*. Clinical variables were *relapse* and *GvHD,* subcategorised as *acute (aGvHD)* or *chronic* *(cGvHD)*. Per 2014 NIH and 2016 Mount Sinai Acute GvHD International Consortium criteria, overall GvHD severity scores of 1 or 2 were considered *mild–moderate* [2, 3], with any higher scores considered *severe* [2, 3]. Data on a/cGvHD point prevalences, i.e. percentage of diagnosed a/cGvHD patients at a measurement time point, were captured on nine predefined clinical data collection days + 30, + 60, + 90, + 120, + 150, + 180, + 240, + 300, + 365 (± 7 days each) [27].

The SMILeApp symptom checklist was derived from the PRO-CTCAE®-based 43-item PROVIVO symptom questionnaire [33], the only validated instrument for symptom distress in alloSCT patients, demonstrating excellent content validity (Scale-CVI: 0.94; Item-CVI median 1.00, range 0.75–1.00) [33]. For remote patient-reported SMILeApp monitoring, it needed to be reduced to its most relevant items (item content validity range: 0.89–1.00) by expert rating. This left 10 items on symptom distress and three on symptom occurrence [27]. Following usability testing, the PROVIVO 5-point (0–4), 7-day scale[33] was adapted to an 11-point (0–10), same-day scale [29]. A previous validation study indicated comparability of the two response scales [34].

Finally, the SMILeApp-reported *symptom distress variables* included (1) *pain*, (2) *signs of bleeding*, (3) *nausea*, (4) *mouth/throat sores*, (5) *dyspnea* (shortness of breath), (6) *dysuria* (painful or burning urination), (7) *fatigue* (tiredness, exhaustion, or lack of energy), (8) *dysphagia* (difficulty swallowing), (9) *decreased appetite*, and (10) *cough* [27]. Using the SMILeApp interface’s symptom checklist, patients first ticked the symptoms currently distressing them (no tick = no distress/0). For each ticked symptom, they scored their distress on a scale from 1 (minimal) to 10 (maximal). Anticipatory distress was not assessed. Patients were regularly reminded to enter SMILeApp data at least weekly.

### Data analyses

For the study aim of describing symptom distress evolution, we first calculated each symptom’s distress point prevalences, the medians of co-occurring distressing symptoms, and summary statistics of distress scores. All of these were captured initially at day −10 pre-alloSCT, then at each post-alloSCT measurement, i.e. days + 30, + 60, + 90, + 120, + 150, + 180, + 240, + 300 (± 7 days each) and + 365 (± 12 days, as patients reported less frequently towards the intervention period’s end). Second, we plotted first-year distress score trajectories, using thin plate regression splines. Third, we determined the entire duration of each symptom’s distress (days) and distress persistence > 30 days uninterrupted (percentages of patients).

For the second study aim to explore symptom distress evolution in view of GvHD, gender and age group (18–64 or ≥ 65 years), we applied boxplot faceting to the three most dominant and longest-lasting symptom distresses. Boxplot faceting descriptively partitions complex data into subplots for a visual comparison of subgroup trajectories. Therefore, no inferential analysis was conducted; sample size calculations are inapplicable. Finally, we calculated GvHD point prevalences for each measurement time point and explored parallel-evolving trends with symptom distress point prevalences.

To address bias, we began by analysing all missing values, i.e. no SMILeApp entry for > 7 days. We tested whether logistic regression could predict types of missing values by regressing each patient-related and clinical variable onto the missing values. Significance level was set at 0.05 and corrected for multiple testing using the Benjamini–Hochberg method [35]. Missing at random or missing not at random hypotheses were accepted or rejected according to significance levels.

We then used the exact Fisher test to identify between-centre differences regarding patient-related and clinical variables, study duration, SMILeApp entry counts, or missing value counts. Further, by calculating centre-level intra-class coefficients for all symptom distress variables, we tested for relevant centre-specific symptom distress differences. Statistical procedures were performed using R v4.2.1© and dplyr v1.1.1, ggplot2 v 3.4.1, ICC.Sample.Size v1.0, mgcv v1.8–42 software packages [36–39].

## Results

### Sample characteristics

Our descriptive analysis included 67 valid and complete patient datasets with 15 789 reporting days regarding symptom distress. Table 1 presents this sub-study’s sample characteristics.

**Table 1:** Sub-Study Sample Characteristics

Characteristics	Total Sample of Sub-study (*N* = 67 Patients)	
	***n***	***%***
**Data at Baseline**		
Gender		
Male	43	64.2
Female	24	35.8
Age group		
< 65 years of age	49	73.1
≥ 65 years of age	18	26.9
Living alone	12	17.9
Diagnoses		
MDS^1^, MPN^2^	23	34.3
ALL^3^, AML^4^	33	49.3
BCL^5^, TCL^6^, NHL^7^, MM^8^	9	13.4
SAA^9^, SM^10^	2	3.0
**Data at 1-Year Post Allogeneic Stem Cell Transplantation**		
1-Year Complications		
Graft-versus-Host-Disease in total	49	73.1
Only mild–moderate^11^ Graft-versus-Host-Disease	24	35.8
Severe^12^ Graft-versus-Host-Disease	25	37.3
Relapse	19	28.4
Reporting Regularity	***Mdn***	***IQR***
Period of post-transplant days	359.0	339.5–365.0
Count of SMILeApp entries	251.0	160.5–324.5
Count of missing values (< 1 SMILeApp entry/week)	3.0	1.0–6.0

*Notes, abbreviations.* Patient characteristics from subsample of main SMILe study [6, 27]. Diagnoses subgroups according to WHO classification [40, 41]: ^1^MyeoloDysplastic Syndromes, ^2^MyeloProliferative Neoplasms, ^3^Acute Lymphatic Leukemias, ^4^Acute Myeloic Leukemias, ^5^B-Cell-Lymphomas, ^6^ T-Cell-Lymphomas, ^7^Non-Hodgkin-Lymphomas, ^8^Multiple Myelomas, ^9^Aplastic Anaemia, ^10^Systemic Mastocytosis. Graft-versus-Host subgroups according to 2016 Mount Sinai Acute GvHD International Consortium criteria and 2014 National Institutes of Health criteria [2, 3]: ^11^overall severity scores of 1 or 2, ^12^overall severity scores ≥ 3

Regarding bias, both missing at random and missing not at random hypotheses were rejected. We found no significant between-centre differences. Centre-level intra-class coefficients were ≤ 0.12 for all symptoms; therefore, results are presented together.

### Aim 1: Symptom distress evolution

#### Symptom distress point prevalences

Table 2 shows symptom distress point prevalences of the patients with ≥ 1 SMILeApp entry/week according to the predefined measurement time points.

**Table 2:** Point-Prevalences of Graft-versus-Host Disease and of Symptom Distress

**All Patients in this Sub-Study**																				
** Number of All Patients**	**67**		**67**		**65**		**63**		**63**		**63**		**62**		**58**		**57**		**55**	
Days pre-/post-alloSCT^1^	−10 (± 7)		+ 30 (± 7)		+ 60 (± 7)		+ 90 (± 7)		+ 120 (± 7)		+ 150 (± 7)		+ 180 (± 7)		+ 240 (± 7)		+ 300 (± 7)		+ 365 (± 7)	
	*n*	*%*	*n*	*%*	*n*	*%*	*n*	*%*	*n*	*%*	*n*	*%*	*n*	*%*	*n*	*%*	*n*	*%*	*n*	*%*
**Graft-versus-Host Disease**	0	0.0	18	26.9	23	35.4	18	28.6	22	34.9	19	30.1	16	25.8	16	27.6	14	24.6	13	23.6
**Only the Reporting Patients with ≥ 1 SMILeApp Entry/Week According to the Measurement Time Point**																				
** Number of Reporting Patients**	**67**		**65**		**61**		**61**		**62**		**59**		**57**		**56**		**54**		**46**	
Days pre-/post-alloSCT^1^	−10 (± 7)		+ 30 (± 7)		+ 60 (± 7)		+ 90 (± 7)		+ 120 (± 7)		+ 150 (± 7)		+ 180 (± 7)		+ 240 (± 7)		+ 300 (± 7)		+ 365 (± 12)	
	*n*	*%*	*n*	*%*	*n*	*%*	*n*	*%*	*n*	*%*	*n*	*%*	*n*	*%*	*n*	*%*	*n*	*%*	*n*	*%*
** Graft-vs-Host-Disease**	0	0.0	18	27.7	20	32.8	17	27.9	21	33.9	17	28.8	15	26.3	16	28.6	14	25.9	12	26.1
Acute Form	0	0.0	18	27.7	17	27.9	15	24.6	14	22.6	11	18.6	5	8.8	3	5.4	1	1.9	0	0.0
Chronic Form	0	0.0	0	0.0	3	4.9	2	3.3	7	11.3	6	10.2	10	17.5	13	23.2	13	24.1	12	26.1
Symptom Distress from																				
Fatigue	14	20.9	50	76.9	41	67.2	36	59.0	33	53.2	29	49.2	29	50.9	27	48.2	27	50.0	23	50.0
Decreased Appetite	2	3.0	37	56.9	29	47.5	27	44.3	23	37.1	21	35.6	20	35.1	16	28.6	12	22.2	10	21.7
Pain	6	9.0	31	47.7	24	39.3	20	32.8	18	29.0	15	25.4	21	36.8	16	28.6	14	25.9	12	26.1
Nausea	0	0.0	30	46.2	23	37.7	18	29.5	17	27.4	10	16.9	10	17.5	10	17.9	6	11.0	4	8.7
Dyspnea	7	10.4	15	23.1	9	14.8	8	13.1	8	12.9	10	16.9	8	14.0	9	16.1	9	16.7	6	13.0
Cough	1	1.5	12	18.5	11	18.0	9	14.8	9	14.5	11	18.6	9	15.8	7	12.5	5	9.3	9	19.6
Sores	1	1.5	8	12.3	8	13.1	5	8.2	7	11.3	8	13.6	7	12.3	7	12.5	7	13.0	6	13.0
Dysuria	0	0.0	7	10.8	8	13.1	7	11.5	6	9.7	6	10.2	2	3.5	5	8.9	4	7.4	1	2.2
Dysphagia	0	0.0	8	12.3	8	13.1	5	8.2	2	3.2	6	10.2	2	3.5	3	5.4	2	3.7	0	0.0
Bleeding Signs	0	0.0	4	6.2	4	6.6	3	4.9	3	4.8	5	8.5	4	7.0	1	1.8	1	1.9	2	4.3

*Notes, abbreviations*. Identical numbers of patients in subgroups ≠ identical patients. ^1^alloSCT = allogeneic stem cell transplantation

The six symptom distresses affecting most post-alloSCT patients over the study period were fatigue (48.2–76.9%), decreased appetite (21.7–56.9%), pain (25.4–47.7%), nausea (8.7–46.2%), dyspnea (13.0–23.1%), and cough (9.3–19.6%). Fatigue, decreased appetite, and pain affected > 20% of patients at every post-alloSCT measurement time point. All three were lowest pre-alloSCT (20.9%, 3.0%, 9.0%), and peaked at day + 30 (76.9%, 56.9%, 47.7%), decreasing only moderately until post-alloSCT day + 365 (respectively, 50.0%, 21.7%, 26.1%). Fatigue and pain distress point prevalences remained stable from day + 150.

Nevertheless, most patients were only distressed by small numbers of symptoms simultaneously: co-distressing symptoms’ *median (IQR)* value peaked at 2 (1–3) on day + 30, then decreased permanently to 1 (0–2) from day + 120 – matching pre-alloSCT values.

#### Symptom distress score trajectories

**Fig. 3:**
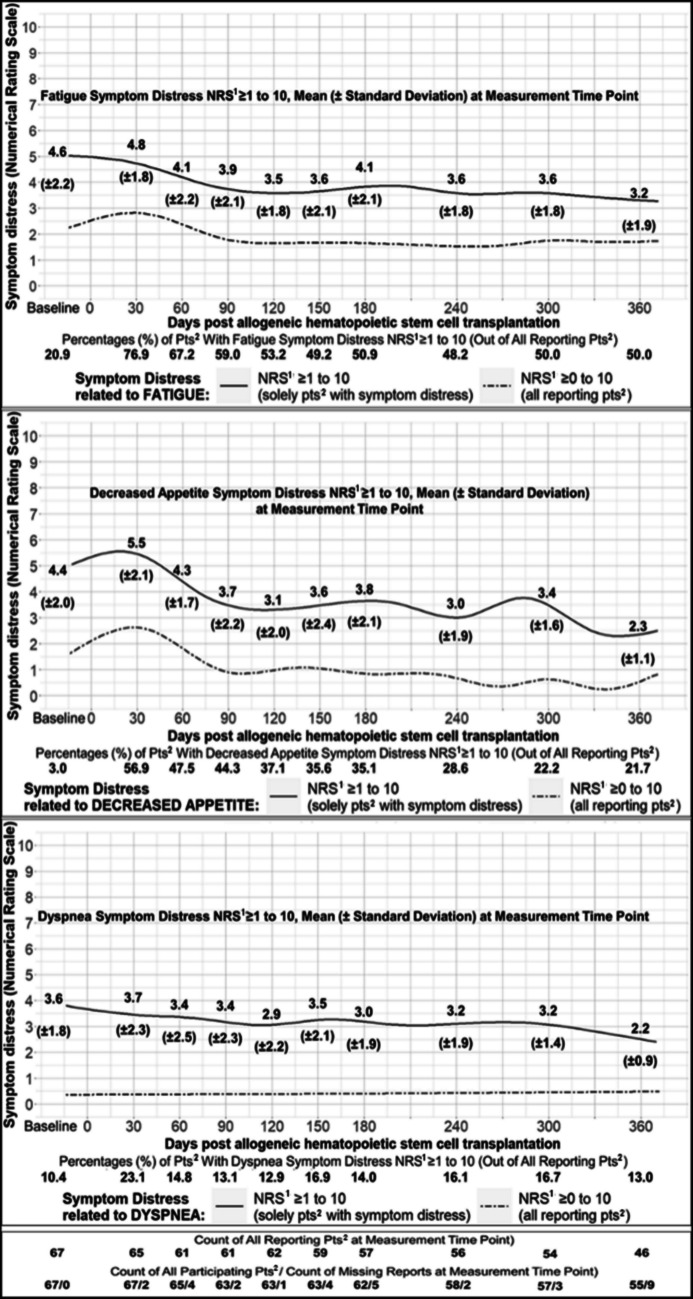
Distress Score Trajectories Related to Fatigue, Decreased Appetite, and Dyspnea. *Notes, abbreviations*. Trajectories visualised by Generalised Additive Modelling using thin plate regression splines. Means (standard deviations) are additionally presented. ^1^NRS = Score of Numerical Rating Scale, ^2^pts = patients

**Fig. 4:**
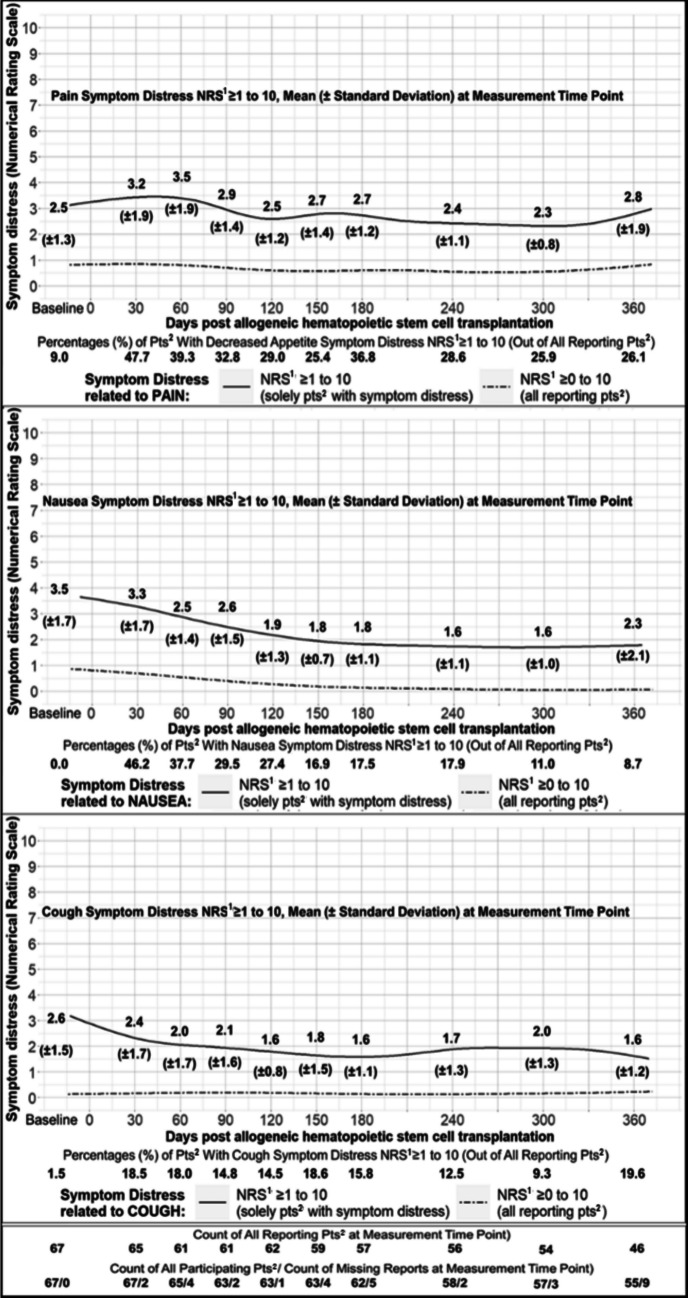
Distress Score Trajectories Related to Pain, Nausea, and Cough. *Notes, abbreviations*. Trajectories visualised by Generalised Additive Modelling using thin plate regression splines. Means (standard deviations) are additionally presented. ^1^NRS = Score of Numerical Rating Scale, ^2^pts = patients

To visualise trajectories of the six prevailing symptom-related distresses (fatigue, decreased appetite, pain, nausea, dyspnea, cough), we plotted two splines for each: one including only patients reporting symptom distress (scores 1–10), and one including patients who also reported no symptom distress on some days (scores 0–10). As splines differ from line charts of discrete means, *mean* (*SD)* scores were additionally calculated for each measurement time point. Figures 3 and 4 depict the prevailing symptoms' distress score trajectories and *means* (*SD*). The trajectories of fatigue, decreased appetite, and dyspnea had the highest *mean* symptom distress scores, all of which peaked at day + 30. From day + 150 until day + 300, fatigue, decreased appetite, and dyspnea all remained distressing with *mean* scores ≥ 3.0.

#### Symptom distress duration and persistence

*Median* (*IQR*) symptom distress duration in days was 87 (36–160) for nausea, 114 (46–202) for decreased appetite, 154 (67–255) for fatigue, 160.5 (88–265) for cough, 165.5 (75–253) for pain, and 181 (88–275) for dyspnea. In 34.3% of the 67 patients, days with and without symptom distress alternated throughout the entire duration. Overall, though, 65.7% of patients reported ≥ 1 period of uninterrupted symptom distress persisting > 30 days, primarily related to fatigue (52.2%), decreased appetite (43.3%), nausea (19.4%), dyspnea (14.9%), and pain (13.4%).

### Aim 2: Symptom distress evolution in view of GvHD, gender, age group

#### Boxplot faceting

**Fig. 5:**
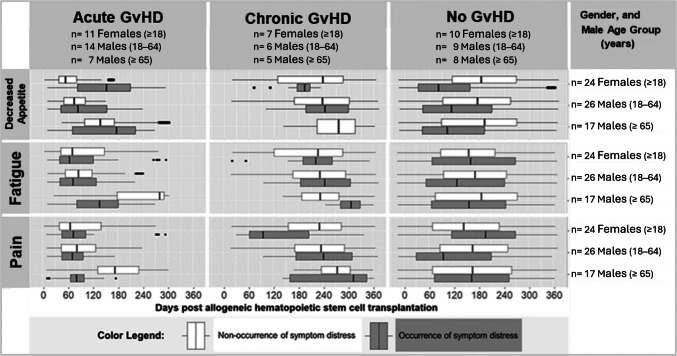
Persistence of Distress Related to Decreased Appetite, Fatigue, and Pain Post-Transplant, Faceted by Graft-versus-Host-Disease (GvHD), Gender and Age. *Notes, abbreviations.* Non-occurrence of symptom distress corresponded to a score of 0, and symptom distress to a score of ≥ 1 to 10. The boxplots visualise *range*, *minimum, maximum, IQR, median* of each symptom’s distress duration (days). Time periods with (1) solely the grey box represent persistent symptom distress without interruption, (2) grey and white boxes represent an alternation of days with and without symptom distress, and (3) solely the white box represent uninterrupted non-occurrence of symptom distress. When the boxplot range does not correspond to the whole time period, there were periods without patients in the according subgroup. Four females and males had both acute and chronic GvHD

In Fig. 5, we visualised all long (> 30-day) periods of uninterrupted persistent symptom distress from fatigue, decreased appetite, and pain faceted to indicate GvHD, gender, and age subgroups. We formed subgroups by age at 1-year post-alloSCT: (a) 26 males aged 18–64, (b) 17 males aged ≥ 65 (senior males), and (c) 24 females aged ≥ 18. With only four women aged ≥ 65, female age subgrouping was not performed.

While all subgroups reported early periods of persistent fatigue-related symptom distress ending ≈ day + 100, senior male aGvHD patients’ fatigue-related reports ended later ≈ day + 180. Late-onset periods were reported by the male cGvHD patients (≈ days + 280 to + 330), and also by seven of 10 female non-GvHD patients (≈ days + 215 to + 270).

Regarding persistent pain-related symptom distress, senior male aGvHD patients and male non-GvHD patients aged 18–64 reported early long persistence periods ending ≈ day + 100. However, female cGvHD patients reported such periods ending later ≈ day + 160. Late-onset periods were reported by senior male cGvHD patients (≈ days + 160 to + 240 and ≈ + 310 to + 350), and also by five of the 10 female non-GvHD patients (≈ days + 220 to + 280).

Regarding persistent decreased appetite-related symptom distress, all non-GvHD subgroups' early long-persistent periods ended ≤ day + 120. In the aGvHD patients, these periods started and ended later, and persisted longest in females (≈ days + 85 to + 210).

#### GvHD point prevalences

Table 2 details point prevalences of GvHD and different symptom-related distresses. Overall GvHD point prevalences fluctuated. The highest GvHD prevalences were at days + 60 (35.4%) and + 120 (34.9%); the lowest (23.6%) at day + 365. Whereas aGvHD point prevalences decreased steadily from day + 90, cGvHD point prevalences slowly increased from day + 150. Decreasing aGvHD point prevalences paralleled decreasing symptom distress point prevalences related to nausea and decreased appetite from day + 90.

The fluctuating overall GvHD point prevalences paralleled fluctuating symptom-related distress point prevalences regarding fatigue (49.2%–50.0%), pain (25.4%–26.1%), dyspnea (16.9%–13.0%), and mouth/throat sores (13.6–13.0%) from day + 150 to + 365.

## Discussion

This SMILe project sub-study is the first longitudinal observational study providing prospective data on first-year post-alloSCT symptom distress evolution. We analysed 15 789 days with regular alloSCT patient entries from 67 patients on 10 relevant symptoms. We also particularly looked at how symptom distress evolved in view of GvHD, gender, and age group.

Patients experienced substantial multi-domain, multidimensional symptom-related distress with fatigue, decreased appetite, pain, and dyspnea showing (I) the highest symptom distress point prevalences, (II) high-scoring (≥ 3) symptom distress trajectories, (III) months-long duration, and (IV) long (> 30-day) periods of uninterrupted symptom distress persistence post-alloSCT. Parallel-evolving trends of GvHD and symptom distress point prevalences suggest that concurrent aGvHD and cGvHD may influence longitudinal symptom distress evolution.

The comparability of our findings regarding fatigue-, decreased appetite-, pain-, and dyspnea-related distress could indicate potential interdependent associations between them. In this respect, a meta-analysis covering 5 630 haematological and solid cancer patients showed significant correlations between cancer-related fatigue's occurrences and symptom distress related to lack of appetite, pain, and dyspnea (respective mean effect sizes d = 0.65, d = 0.60 and d = 0.45) [42]. Furthermore, a longitudinal study identified a symptom cluster comprising a stable co-occurrence of fatigue, appetite loss, and dyspnea until year + 5 post-alloSCT [14]. However, potential interdependencies among post-alloSCT symptoms have not yet been investigated. This will become a feasible next step when remote symptom-monitoring systems are more widely adopted.

Our results support previous findings that fatigue [18–21], decreased appetite [18, 20–22], and dyspnea [22] are among the most distressing symptoms in the first months post-alloSCT. However, heterogeneous measurement time points and questionnaires limited consistency across previous studies. Using SMILe study's remote patient-reported monitoring, we discovered how persistent and high-scoring (≥ 3.0) their related distress can be for post-alloSCT patients until at least day + 300. Our findings show symptom distress point prevalences (21.7%–76.9%) for first-year post-alloSCT fatigue, decreased appetite and pain lower than previously reported (37.0%–94.0%) [19, 22]. Several factors may explain these differences, including variations in measurement time points, contextual influences, and healthcare structures. Another explanation is that our study assessed symptom distress in SMILe-ICM patients, a sample receiving already enhanced follow-up with self-management support, early detection, and optimised symptom management, potentially reducing symptom distress [27, 28]. Our findings may inform future research in symptom management and potentially risk stratification, as supported by a meta-analysis of 44 030 cancer patients demonstrating the prognostic value of patient-reported fatigue and pain for overall survival [43]. Importantly, these results underscore the need for healthcare systems to implement systematic remote symptom monitoring post-alloSCT to enable timely interventions, optimise recovery, and support individualised, risk-stratified care.

Regarding patient-related factors, senior male aGvHD and female GvHD patients experienced exceptionally long persistent distress (until ≈ 160 to 180 days post-alloSCT); while male cGvHD and female non-GvHD patients reported second late-onset long periods of decreased appetite-, fatigue-, and pain-related distress persistence. These subgroup patterns may reflect underlying mechanisms such as chemotherapy-induced menopause in female non-GvHD patients or GvHD-related inflammation, compounded by hormonal deficiencies in GvHD patients; however, these explanations remain hypothetical.

### Limitations

This study's strengths include its prospective longitudinal observational design combined with the high number of at least weekly electronic entries from two European centres. However, several limitations should be considered. First, the small sample size (*N* = 67) significantly reduces generalisability, particularly in subgroup analyses, where visual exploration using boxplot faceting is constrained by small numbers. Second, the SMILeApp symptom distress questionnaire's reliability and construct validity remain to be formally evaluated by systematic psychometric testing. The questionnaire was adapted for daily screening and rapid team response. This may notably limit its suitability for comprehensive symptom distress research. Third, we could not analyse the SMILe intervention effect on our findings because no symptom data were available for the main SMILe study control group. Finally, broader generalisability is limited both by the numbers of participating patients and centres, and the influence of the SARS-COVID19 pandemic on data collection, as related public hygiene measures might have significantly reduced the prevalence of respiratory infections and symptoms. Consequently, the study results are exploratory and require validation in larger, more representative cohorts. Despite these limitations, the study provides valuable initial insights into symptom distress evolution and associated care needs throughout the first year post-alloSCT. We also identify patient groups potentially at heightened risk for persistent symptom distress.

### Implications

Throughout patients’ first post-alloSCT year, they are challenged by multi-domain, multidimensional distress and, especially in some subgroups, that distress’s persistence. To minimise the risks regarding reduced QoL and ongoing health impairments [19, 22–24], both must be targeted directly in clinical practice. For example, mild–moderate cGvHD has a lower relapse risk and improved long-term prognosis [44], making treating physicians more likely to accept it. Our findings suggest that in addition to considering cGvHD severity, patient-centred cGvHD management benefits from regular patient-reported monitoring, especially for fatigue-, decreased appetite-, dyspnea-, and pain-related symptom distress. Alleviative or supportive interventions must consider the accompanying symptom distress's physical and psychosocial consequences, and their impacts on patients' daily activities. Continuous remote monitoring and an integrated approach should be considered for long-term symptom distress management.

Further confirmatory and longitudinal research is needed – first, to inform multiple-symptom management by clarifying whether and how fatigue, decreased appetite, pain, and dyspnea form a stable *symptom distress cluster* [9, 45]; second, to investigate associations with health outcomes. Third, interventions against fatigue-, decreased appetite-, pain-, and dyspnea-related distress with effects across ≥ two symptoms [45] should be combined and tested to determine which combinations best alleviate multiple symptoms’ distress across the selected domains. Fourth, studies will be necessary to clarify impacts of patient-related factors, e.g. GvHD, gender, age, and relapse regarding the evolution of exceptional symptom distress. Finally, interventions incorporating purpose-built coping strategies might need to be tailored to specific patient subgroups, their contexts, their most prevalent symptoms, and the distress scores associated with long-term symptom persistence.

## Conclusion

This SMILe sub-study provides the first prospective longitudinal observational data on symptom distress evolution throughout the first year post-alloSCT, in view of GvHD, gender, and age group. Symptom distress related to fatigue, decreased appetite, pain, and dyspnea was most persistent and impactful, warranting further investigation. These symptoms appear to be key for care coordination and risk stratification, highlighting the need for personalised, symptom-focused strategies in post-alloSCT survivorship care. While preliminary, these results offer a foundation for hypothesis generation and underscore the value of implementing electronic patient-related outcomes in clinical practice.

Further research is essential, particularly regarding subgroups affected by persistent symptom distress. Gender- and age-tailored GvHD management could help disrupt symptom distress persistence, while regular remote patient-reported monitoring facilitates timely care adjustments. Building on this study's longitudinal analyses, both confirmatory and exploratory research is needed to inform strategies that optimise post-alloSCT symptom distress management, alleviate patient burden, and improve long-term outcomes.

## Data Availability

No individual patient data sharing to third parties is possible. Access to aggregated data is available upon reasonable request to the corresponding author.

## References

[1] Styczyński J, Tridello G, Koster L et al (2020) Death after hematopoietic stem cell transplantation: changes over calendar year time, infections and associated factors. Bone Marrow Transplant 55:126–136. 10.1038/s41409-019-0624-z 31455899 10.1038/s41409-019-0624-z PMC6957465

[2] Harris AC, Young R, Devine S et al (2016) International, multicenter standardization of acute graft-versus-host disease clinical data collection: a report from the Mount Sinai Acute GVHD International Consortium. Biol Blood Marrow Transplant 22:4–10. 10.1016/j.bbmt.2015.09.001 26386318 10.1016/j.bbmt.2015.09.001 PMC4706482

[3] Jagasia MH, Greinix HT, Arora M et al (2015) National Institutes of Health consensus development project on criteria for clinical trials in chronic graft-versus-host disease: I. The 2014 diagnosis and staging working group report. Biol Blood Marrow Transplant 21:389-401.e1. 10.1016/j.bbmt.2014.12.001 25529383 10.1016/j.bbmt.2014.12.001 PMC4329079

[4] Mahmoudjafari Z, Bhatt V, Galvin J et al (2025) Impact of cytopenias and early versus late treatment with ruxolitinib in patients with steroid-refractory acute or chronic graft-versus-host disease. Bone Marrow Transplant 60:69–78. 10.1038/s41409-024-02445-6 39506073 10.1038/s41409-024-02445-6 PMC11726446

[5] Gunduz M, Atilla E, Atilla PA et al (2019) Early initiation of extracorporeal photochemotherapy increases response for chronic graft versus host disease following steroid failure. Transfusion Clinique et Biologique 26:32–37. 10.1016/j.tracli.2018.03.005 29655590 10.1016/j.tracli.2018.03.005

[6] Leppla L, Kaier K, Schmid A et al (2024) Evaluating the cost, cost-effectiveness and survival of an eHealth-facilitated integrated care model for allogeneic stem cell transplantation: Results of the German SMILe randomized, controlled implementation science trial. Eur J Oncol Nurs Off J Eur Oncol Nurs Soc 74:102740. 10.1016/j.ejon.2024.102740 10.1016/j.ejon.2024.102740 39591883

[7] Basch E, Hudson K, Rocque G (2023) Implementation of electronic patient-reported outcomes for symptom monitoring during cancer treatment: the importance of getting it right. J Comp Eff Res 12:e230157. 10.57264/cer-2023-0157 37961992 10.57264/cer-2023-0157 PMC10734319

[8] Lizán L, Pérez-Carbonell L, Comellas M (2021) Additional value of patient-reported symptom monitoring in cancer care: a systematic review of the literature. Cancers 13:4615. 10.3390/cancers13184615 34572842 10.3390/cancers13184615 PMC8469093

[9] Miaskowski C, Barsevick A, Berger A et al (2017) Advancing symptom science through symptom cluster research: expert panel proceedings and recommendations. JNCI J Natl Cancer Inst 109:djw253. 10.1093/jnci/djw253 28119347 10.1093/jnci/djw253 PMC5939621

[10] Fu MR (2004) An integrated approach to an analysis of symptom management in patients with cancer. Oncol Nurs Forum 31(1):65–70. 10.1188/04.ONF.65-70 14722589 10.1188/04.ONF.65-70

[11] Sidana S, Dueck AC, Thanarajasingam G et al (2022) Longitudinal Patient Reported Outcomes with CAR-T Cell Therapy vs Autologous and Allogeneic Stem Cell Transplant. Transplant Cell Ther 28:473–482. 10.1016/j.jtct.2022.05.004 35550440 10.1016/j.jtct.2022.05.004 PMC9357185

[12] Bryant AL, Coffman E, Phillips B et al (2020) Pilot randomized trial of an electronic symptom monitoring and reporting intervention for hospitalized adults undergoing hematopoietic stem cell transplantation. Support Care Cancer 28:1223–1231. 10.1007/s00520-019-04932-9 31222392 10.1007/s00520-019-04932-9 PMC6923608

[13] Palmer J, Kosiorek HE, Wolschke C et al (2019) Assessment of quality of life following allogeneic stem cell transplant for myelofibrosis. Biol Blood Marrow Transplant 25:2267–2273. 10.1016/j.bbmt.2019.07.001 31288096 10.1016/j.bbmt.2019.07.001 PMC8114229

[14] Esser P, Kuba K, Scherwath A et al (2017) Stability and priority of symptoms and symptom clusters among allogeneic HSCT patients within a 5-year longitudinal study. J Pain Symptom Manage 54:493–500. 10.1016/j.jpainsymman.2017.07.012 28711754 10.1016/j.jpainsymman.2017.07.012

[15] Frödin U, Lotfi K, Fomichov V et al (2015) Frequent and long-term follow-up of health-related quality of life following allogeneic haematopoietic stem cell transplantation. Eur J Cancer Care (Engl) 24:898–910. 10.1111/ecc.12350 26156141 10.1111/ecc.12350

[16] Cohen MZ, Rozmus CL, Mendoza TR et al (2012) Symptoms and quality of life in diverse patients undergoing hematopoietic stem cell transplantation. J Pain Symptom Manage 44:168–180. 10.1016/j.jpainsymman.2011.08.011 22699091 10.1016/j.jpainsymman.2011.08.011 PMC4270122

[17] Andersson I, Ahlberg K, Stockelberg D et al (2009) Health-related quality of life in patients undergoing allogeneic stem cell transplantation after reduced intensity conditioning versus myeloablative conditioning. Cancer Nurs 32:325–334. 10.1097/NCC.0b013e31819b5c81 19444087 10.1097/NCC.0b013e31819b5c81

[18] Jarden M, Nelausen K, Hovgaard D et al (2009) The effect of a multimodal intervention on treatment-related symptoms in patients undergoing hematopoietic stem cell transplantation: a randomized controlled trial. J Pain Symptom Manage 38:174–190. 10.1016/j.jpainsymman.2008.09.005 19345060 10.1016/j.jpainsymman.2008.09.005

[19] Bevans MF, Mitchell SA, Marden S (2008) The symptom experience in the first 100 days following allogeneic hematopoietic stem cell transplantation (HSCT). Support Care Cancer 16:1243–1254. 10.1007/s00520-008-0420-6 18322708 10.1007/s00520-008-0420-6 PMC2885854

[20] Larsen J, Nordström G, Ljungman P, Gardulf A (2004) Symptom occurrence, symptom intensity, and symptom distress in patients undergoing high-dose chemotherapy with stem-cell transplantation. Cancer Nurs 27:55–64. 10.1097/00002820-200401000-00007 15108952 10.1097/00002820-200401000-00007

[21] Larsen J, Nordström G, Ljungman P, Gardulf A (2007) Factors associated with poor general health after stem-cell transplantation. Support Care Cancer 15:849–857. 10.1007/s00520-006-0200-0 17205276 10.1007/s00520-006-0200-0

[22] Eriksson LV, Holmberg K, Lundh Hagelin C et al (2023) Symptom burden and recovery in the first year after allogeneic hematopoietic stem cell transplantation. Cancer Nurs 46:77–85. 10.1097/NCC.0000000000001077 35283470 10.1097/NCC.0000000000001077

[23] Bevans MF, Mitchell SA, Barrett J et al (2014) Symptom distress predicts long-term health and well-being in allogeneic stem cell transplant survivors. Biol Blood Marrow Transplant J Am Soc Blood Marrow Transplant 20:387–395. 10.1016/j.bbmt.2013.12.001 10.1016/j.bbmt.2013.12.001 PMC3962950 24355521

[24] Kelly DL, Lyon DE, Ameringer SA et al (2015) Symptoms, cytokines, and quality of life in patients diagnosed with chronic Graft-Versus-Host disease following allogeneic hematopoietic stem cell transplantation. Oncol Nurs Soc 42:265–275. 10.1188/15.ONF.265-275 10.1188/15.ONF.265-275 25901378

[25] Henoch I, Sawatzky R, Falk H et al (2014) Symptom distress profiles in hospitalized patients in Sweden: a cross-sectional study. Res Nurs Health 37:512–523. 10.1002/nur.21624 25308151 10.1002/nur.21624

[26] Valenta S, Ribaut J, Leppla L et al (2023) Context-specific adaptation of an eHealth-facilitated, integrated care model and tailoring its implementation strategies—a mixed-methods study as a part of the SMILe implementation science project. Front Health Serv. 10.3389/frhs.2022.977564 36925799 10.3389/frhs.2022.977564 PMC10012712

[27] De Geest S, Valenta S, Ribaut J et al (2022) The SMILe integrated care model in allogeneic stem cell transplantation facilitated by eHealth: a protocol for a hybrid effectiveness-implementation randomised controlled trial. BMC Health Serv Res 22:1067. 10.1186/s12913-022-08293-8 35987671 10.1186/s12913-022-08293-8 PMC9392360

[28] Leppla L, Schmid A, Valenta S et al (2021) Development of an integrated model of care for allogeneic stem cell transplantation facilitated by eHealth—the SMILe study. Support Care Cancer 29:8045–8057. 10.1007/s00520-021-06328-0 34224016 10.1007/s00520-021-06328-0 PMC8550349

[29] Leppla L, Hobelsberger S, Rockstein D et al (2021) Implementation science meets software development to create eHealth components for an integrated care model for allogeneic stem cell transplantation facilitated by eHealth: the SMILe study as an example. J Nurs Scholarsh 53:35–45. 10.1111/jnu.12621 33348461 10.1111/jnu.12621

[30] Ribaut J, Leppla L, Teynor A et al (2020) Theory-driven development of a medication adherence intervention delivered by eHealth and transplant team in allogeneic stem cell transplantation: the SMILe implementation science project. BMC Health Serv Res 20:827. 10.1186/s12913-020-05636-1 32878623 10.1186/s12913-020-05636-1 PMC7465386

[31] Ribaut J, Geest SD, Leppla L et al (2022) Exploring Stem Cell Transplanted Patients&rsquo; Perspectives on Medication Self-Management and Electronic Monitoring Devices Measuring Medication Adherence: A Qualitative Sub-Study of the Swiss SMILe Implementation Science Project. Patient Prefer Adherence 16:11–22. 10.2147/PPA.S337117 35023905 10.2147/PPA.S337117 PMC8747798

[32] Leppla L, Mielke J, Kunze M et al (2020) Clinicians and patients perspectives on follow-up care and eHealth support after allogeneic hematopoietic stem cell transplantation: a mixed-methods contextual analysis as part of the SMILe study. Eur J Oncol Nurs. 10.1016/j.ejon.2020.101723 32062362 10.1016/j.ejon.2020.101723

[33] Kirsch M, Mitchell SA, Dobbels F et al (2015) Linguistic and content validation of a German-language PRO-CTCAE-based patient-reported outcomes instrument to evaluate the late effect symptom experience after allogeneic hematopoietic stem cell transplantation. Eur J Oncol Nurs 19:66–74. 10.1016/j.ejon.2014.07.007 25190633 10.1016/j.ejon.2014.07.007

[34] Lee MK, Schalet BD, Cella D et al (2020) Establishing a common metric for patient-reported outcomes in cancer patients: linking patient reported outcomes measurement information system (PROMIS), numerical rating scale, and patient-reported outcomes version of the common terminology criteria for adverse events (PRO-CTCAE). J Patient-Rep Outcomes 4:106. 10.1186/s41687-020-00271-0 33305344 10.1186/s41687-020-00271-0 PMC7728866

[35] Benjamini Y, Hochberg Y (1995) Controlling the false discovery rate: a practical and powerful approach to multiple testing. J R Stat Soc Ser B Methodol 57:289–300. 10.1111/j.2517-6161.1995.tb02031.x

[36] R Core Team (2021) R: A language and environment for statistical computing. R Foundation for Statistical Computing, Vienna, Austria.

[37] Wickham H (2016) ggplot2: Elegant Graphics for Data Analysis. https://ggplot2.tidyverse.org. Accessed 11 June 2023

[38] Wickham H, François R, Henry L, et al (2023) dplyr: A Grammar of Data Manipulation. https://cran.r-project.org/web/packages/dplyr/index.html. Accessed 11 June 2023

[39] Rathbone A, Shaw S, Kumbhare D (2015) ICC.Sample.Size: Calculation of Sample Size and Power for ICC. https://cran.r-project.org/web/packages/ICC.Sample.Size/index.html. Accessed 11 June 2023

[40] Alaggio R, Amador C, Anagnostopoulos I et al (2022) The 5th edition of the World Health Organization classification of Haematolymphoid Tumours: lymphoid neoplasms. Leukemia 36:1720–1748. 10.1038/s41375-022-01620-2 35732829 10.1038/s41375-022-01620-2 PMC9214472

[41] Khoury JD, Solary E, Abla O et al (2022) The 5th edition of the World Health Organization classification of Haematolymphoid Tumours: myeloid and histiocytic/dendritic neoplasms. Leukemia 36:1703–1719. 10.1038/s41375-022-01613-1 35732831 10.1038/s41375-022-01613-1 PMC9252913

[42] Oh HS, Seo WS (2011) Systematic review and meta-analysis of the correlates of cancer-related fatigue. Worldviews Evid Based Nurs 8:191–201. 10.1111/j.1741-6787.2011.00214.x 21342434 10.1111/j.1741-6787.2011.00214.x

[43] Huang RS, Chen D, Benour A et al (2025) Patient-reported outcomes as prognostic indicators for overall survival in cancer: a systematic review and meta-analysis. JAMA Oncol 11:1303–1312. 10.1001/jamaoncol.2025.3153 40932728 10.1001/jamaoncol.2025.3153 PMC12426862

[44] Csanadi M, Agh T, Tordai A et al (2019) A systematic literature review of incidence, mortality, and relapse of patients diagnosed with chronic graft versus host disease. Expert Rev Hematol 12:311–323. 10.1080/17474086.2019.1605288 30955381 10.1080/17474086.2019.1605288

[45] Kwekkeboom K (2020) Guideline-recommended symptom management strategies that cross over two or more cancer symptoms. Oncol Nurs Forum 47(5):498–511. 10.1188/20.ONF.498-511 32830800 10.1188/20.ONF.498-511

